# Mild behavioral impairment is related to frailty in non-dementia older adults: a cross-sectional study

**DOI:** 10.1186/s12877-020-01903-2

**Published:** 2020-11-27

**Authors:** Shaoyi Fan, Ximin Liang, Tianchan Yun, Zhong Pei, Bin Hu, Zahinoor Ismail, Zhimin Yang, Fuping Xu

**Affiliations:** 1 The Second Clinical College of Guangzhou University of Chinese Medicine, 232 East Ring Road, Guangzhou, P. R. China; 2 Department of Neurology, First Affiliated Hospital of Sun Yat-sen University, 58 Zhongshan Second Road, Guangzhou, P. R. China; 3 Division of Translational Neuroscience, Department of Clinical Neurosciences, Hotchkiss Brain Institute, Alberta Children’s Hospital Research Institute, Cumming School of Medicine, University of Calgary, 3330 Hospital Drive NW, Calgary, AB T2N 4N1 Canada; 4 Departments of Psychiatry, Clinical Neurosciences, and Community Health Sciences, Hotchkiss Brain Institute and O’Brien Institute for Public Health, University of Calgary, Calgary, Canada; 5 The Second Affiliated Hospital of Guangzhou University of Chinese Medicine, Guangdong Provincial Hospital of Chinese Medicine, 111 Dade Road, Guangzhou, People’s Republic of China

**Keywords:** Cognitive impairment, Frailty, Mild behavioral impairment, Neuropsychiatric symptoms

## Abstract

**Background:**

Frailty and cognitive decline are highly prevalent among older adults. However, the relationship between frailty and mild behavioral impairment (MBI), a dementia risk syndrome characterized by later-life emergence of persistent neuropsychiatric symptoms, has yet to be elucidated. We aimed to evaluate the associations between MBI and frailty in older adults without dementia.

**Methods:**

In this cross-sectional study, a consecutive series of 137 older adults without dementia in the Anti-Aging Study, recruited from primary care clinics, were enrolled. Frailty was estimated using the Fried phenotype. MBI was evaluated by the Mild Behavioral Impairment Checklist (MBI-C) at a cut-off point of > 8. Cognition was assessed with the Chinese versions of the Montreal Cognitive Assessment (MoCA-BC) and Mini-mental State Examination (MMSE). Multivariable logistic regression was performed to estimate the relationship between MBI and objective cognition with frailty status.

**Results:**

At baseline, 30.7% of the older adults had frailty and 18.2% had MBI (MBI+ status). Multivariable logistic regression analysis demonstrated that compared to those without MBI (MBI- status), MBI+ was more likely to have frailty (odds ratio [OR] = 7.44, 95% CI = 1.49–37.21, *p* = 0.02). Frailty and MBI were both significantly associated with both MMSE and MoCA-BC score (*p* < 0.05).

**Conclusions:**

Both frailty and MBI status were associated with higher odds of cognitive impairment. MBI was significantly associated with an increased risk of having frailty in the absence of dementia. This association merits further study to identify potential strategies for the early detection, prevention and therapeutic intervention of frailty.

## Background

Frailty is a common geriatric condition presenting as a clinical state of decreased physiological reserve, increased vulnerability to death and increased susceptibility to even small stressors [1]. It is associated with an increased risk of adverse health-related outcomes, including falls, disability and mortality [2]. The prevalence of frailty is 3.9 to 51.4% among community-dwelling people aged 60 years and older, and the incidence increases with age [3]. As population aging has become a global phenomenon, frailty has become an emerging public health issue. To date, most definitions have prioritized the physical dimension of frailty, which includes symptoms and signs such as weight loss, muscle weakness, slower gait speed, and sedentary behavior [4]. Frailty has been most commonly operationalized using a phenotypic approach or a deficit accumulation approach [5, 6]. In research, a commonly used approach to capture frailty is the Fried phenotype, which has been extensively tested for its validity [7, 8].

Frailty that combines a range of diverse deficits is increasingly recognized as a fundamental determinant of an individual’s vulnerability or resilience to stressors [9] and has been linked to impaired cognition [10, 11]. Cognitive impairment has been shown to improve the predictive value of frailty, measured using the Fried phenotype, for adverse health outcomes [11]. Various neurocognitive disorders, including late-life cognitive impairment [12, 13], mild cognitive impairment (MCI) [14], dementia [15] and Alzheimer’s disease (AD) [16, 17], have shown associations with frailty. Indeed, frailty moderates the association between AD pathology and the clinical expression of dementia, such that in the presence of frailty, even low AD pathological burden may manifest as dementia [17]. Researchers have also found that frailty and cognitive decline might share common physiological mechanisms, with greater frailty being associated with worse cognition and a faster rate of cognitive decline [18]. Thus, associations between frailty and other risk markers for cognitive decline are warranted.

Similar to frailty, neuropsychiatric symptoms (NPS) have demonstrated associations with cognitive decline and have been linked to known dementia biomarkers, thus also suggesting common underlying mechanisms. The Mayo Clinic Study of Aging reported that the presence of NPS (particularly agitation, apathy, anxiety, irritability or depression) was associated with an increased risk of developing MCI in cognitively normal older adults [19]. More recent evidence from a large sample in the National Alzheimer Coordinating Center dataset demonstrated that in 59% of dementia cases, NPS emerged in advance of cognitive symptoms, including 30% of people who developed AD, reinforcing the notion that later-life onset of NPS can be an early marker of dementia [20]. To operationalize the assessment of NPS as risk markers for dementia, the International Society to Advance Alzheimer’s Research and Treatment developed criteria for mild behavioral impairment (MBI) [21], which is a neurobehavioral syndrome characterized by later-life emergent NPS as an at-risk state for incident cognitive decline and dementia. Although MBI and MCI can co-occur, MBI can also precede MCI, manifesting in older adults with subjective cognitive decline or even normal cognition, in whom MBI has demonstrated an increased risk of cognitive decline and dementia [22–26]. MBI may be the initial manifestation of neurodegeneration for some, and has been connected with known biomarkers for dementia including amyloid beta [27], tau [28, 29], neurofilament light [30], cortical atrophy [31, 32], white matter atrophy [33], and AD risk genes [34, 35]. MBI has also been used in machine learning models to predict neurocognitive diagnostic category 40 months later [36]. These findings suggested that the early recognition of the NPS that constitute MBI may contribute to earlier detection of neurodegeneration, and may represent a clinical entity and premorbid treatment target to explore for intervention strategies to prevent or delay the onset of dementia [37]. The Mild behavioral impairment Checklist (MBI-C) is the validated brief screening instrument developed to capture MBI in accordance with the criteria [38–42].

Frailty, as a substantial moderator in the clinical expression of dementia, could be a predictor of cognitive decline over time [17, 43, 44]. However, the association between frailty and cognition in pre-dementia has yielded mixed results [45–47]. MBI is associated with a significantly faster rate of cognitive decline and progression along the continuum of neurodegenerative pathology compared to late life psychiatric disorders, and compared to those without MBI. Thus predictive value of MBI appears to be early in the neuropathological course of disease, in advance of cognitive impairment for some [22].

Identifying at-risk populations is an important public health issue, in order to explore risk reduction. The possible association between MBI and frailty, both independent risk factors for dementia appearing early in the disease course, should also be further investigated. In this cross-sectional study, we aimed to: 1) determine the prevalence of frailty and of MBI; 2) replicate prior findings linking frailty to worse objective global cognition; 3) determine the association between MBI and global cognition; and 4) assess the relationships between MBI total and domain scores, and frailty, in a primary care sample of older adults with at most mild cognitive impairment. We hypothesized that MBI would predict greater frailty burden.

## Methods

### Participants and setting

**Fig. 1:**
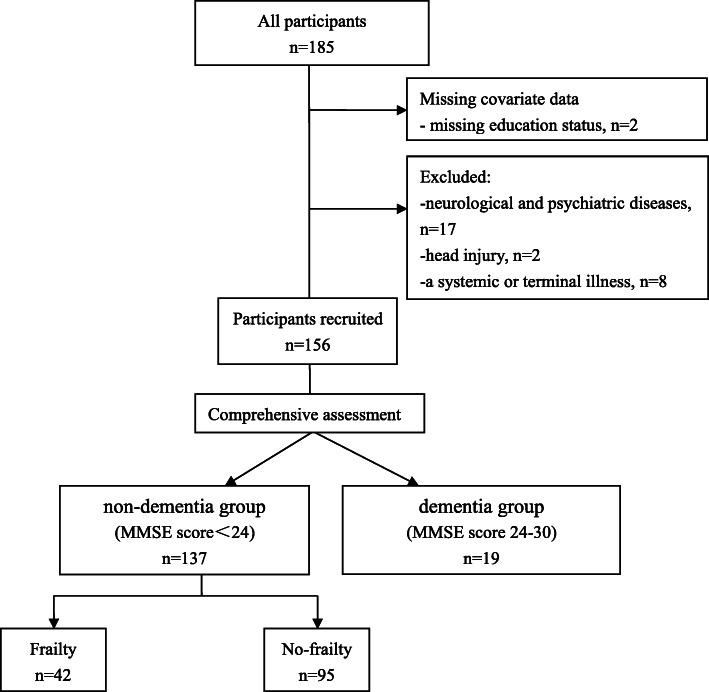
Participant flow chart. Participant inclusion/exclusion criteria. Missing data categories are mutually exclusive

Altogether, a series of 185 volunteers aged 60 or older were consecutively recruited from the course of the Anti-Aging Study, aiming to investigates the association of frailty with health. All subjects were recruited through advertisements at the GPs clinics and Medical Management Centers in Guangzhou (the capital of the Guangdong, South-East of China). 1) aged 60 years or above; 2) ability to speak Chinese; 3) having adequate auditory and visual acuity; and 4) being able to provide wirtten informed consent to participate in the study. All participants were required to complete the eligibility assessment, including an elaborated medical record as well as neuropsychological assessment. Exclusion criteria included the following: 1) those with history of neurological and psychiatric illness,(eg, Alzheimer’s disease, Parkinson’s disease or dementia); 2) brain injury with loss of consciousness lasting at least 5 min or longer; and 3) any multisystemic or potentially life-threatening illness that might affect follow-up interviews. At enrollment, participants were asked to complete a comprehensive evaluation including but not limited to a structured questionnaire that collected demographic, medical record, medication review and clinical characteristics, and frailty assessment, emotional assessment and a neuropsychological assessment. Participants were excluded if they had a cognitive score consistent with dementia, defined as a Mini Mental State Examination (MMSE) cut-off score ≥ 24 [48, 49]. Participants were also excluded if they were missing covariate data (Fig. 1 lists exclusion details).

### Sociodemographic and clinical characteristics

In this study, we evaluated sociodemographic features including sex, age, and education. In addition, nutritional status was determined and classified according to the body mass index (BMI, kg/m^2^), that was calculated as weight (kg) divided by height in meters squared (m^2^) [50]. To identify polypharmacy and multimorbidity, the subjects were asked whether they had a physician-determined diagnosis of hypertension, heart disease, diabetes mellitus, stroke, rheumatic disease, cancer, neurologic disease, osteoporosis (OP), lung disease, urinary incontinence or fecal incontinence. In general, polypharmacy has been defined as concurrent administration of more than 5 long-term medications, while multimorbidity as the coexistence of > 2 chronic conditions [51].

### Emotional assessment

In this study, Anxiety and depression were measured by the Generalized Anxiety Disorder 7-item (GAD-7) scale [52] and the 9-item Patient Health Questionnaire (PHQ-9) [53], respectively. To be specific, the GAD-7 scale determines the frequency of the anxious mood items, which ranges from never (0) to almost every day (3), and the presence of an anxiety symptomatology is defined as a total score of ≥10 [54]. On the other hand, the PHQ-9 scale determines the frequency of the depressed mood items, which ranges from not at all (0) to almost every day (3). The standard cut-off score of 10 or greater maximized the combined sensitivity and specificity in the primary studies [55]. As a result, the GAD-7 score of ≥10 was utilized to be the threshold to indicate the clinically significant anxiety, and the PHQ-9 score ≥ 10 indicates clinically significant depression, respectively [56].

### Frailty assessment

The diagnosis of frailty was based on Fried phenotype in accordance with five indicators: exhaustion, which was based on a self-report questionnaire including two items extracted from the Center for Epidemiology Studies Depression (CES-D) [57]; unintentional weight loss (defined by the weight loss of ≥10 pounds or ≥ 5% of body weight in last year); weak grip strength measured by the dominant grip-strength through the hand grip dynamometer and defined according to an established cutoffs by sex as well as body mass index (BMI); slowed gait speed (speed below an established cutoffs adjusted for sex and height), which was measured by walking time of 15 ft; and low energy expenditure (low level of physical activity over the last 2 weeks, after adjusting for sex), which was predicted by the Minnesota Leisure Time Physical Activity (MLTA) questionnaire [4]. Based on these scores, individuals with 0–2 criteria present were categorized into the no-frailty group and those with 3 or more criteria were in the frailty group [58].

### Neuropsychiatric and neuropsychological assessment

MBI was assessed using the Chinese version of MBI-C developed by Cui [59], a scale established specifically for the community elderly with independent function. The MBI-C [42] includes 34 items classified as five domains: 1) decreased motivation (apathy); 2) emotional dysregulation (symptoms of mood and anxiety disorders); 3) impulse dyscontrol (impulsivity, aggression and agitation); 4) social inappropriateness (impaired social cognition); and 5) abnormal perception or through content (psychotic symptoms, i.e. hallucination and delusions). Only symptoms that were characterized by emergence in later life, representing a change from longstanding patterns of behavior, along with symptoms lasting for at least 6 months, were evaluated as “yes”, with the severity rated (1 to 3 points) [42]. The total scores of MBI-C domain were calculated by adding together the scores of five domains. MBI+ status was based on a total score > 8, its optimal cut-off point for MBI case detection in a primary care population, with good sensitivity and specificity [39].

As part of the objective cognitive assessment, all subjects completed brief objective cognitive screening tools. The Chinese versions of the Montreal Cognitive Assessment (MoCA-BC, Chinese version) [60] together with the Mini-mental State Examination (MMSE, Chinese version) [61] were used to measure global cognitive function. In addition, The MoCA test includes more attention-executive items than the MMSE and was included to be sensitive for mid cognitive impairment, whereas the MMSE was used primarily to screen out dementia [62]. Both MMSE and MoCA were administered randomly for the sake of avoiding the fatigue effect bias. The potential scores range from 0 to 30, with higher values indicating better cognition.

### Statistical analysis

Continuous variables and categorical variables were presented as the mean ± standard deviations (SD) and frequency/percentage respectively. Independent samples t-tests were performed to determine the differences between the frailty and no-frailty groups, with respect to the continuous variables. Chi-square (χ^2^) tests were used to identify the group differences for the categorical variables. As for the MBI-C scale, its total along with the domain-specific questionnaire scores were calculated. The distribution of the scores in MBI-C and the prevalence of MBI diagnosis were determined using frequency and descriptive analyses. In addittion, logistic regression analyse was performed to estimate odds ratios (ORs) with 95% confidence intervals (95% CIs) for the association between frailty status with age, education, depression, MBI and objective cognition. All analyses have been conducted using SPSS statistical analysis software, version 18.0. A two-sided *P*-value of < 0.05 was defined as the level of statistically significance throughout the analysis.

## Results

### Participant characteristics

There were 137 older adults enrolled in this study; the mean age was 69.6 ± 7.6 years, and the age range was 60 to 90 years old. Among these, 94 (68.6%) were female, 21 (15.3%) had a primary school education or lower, 43 (31.4%) had multimorbidity, the presence of more than 2 comorbid conditions, and 27 (19.7%) had polypharmacy (took five or more oral medications daily). Of these enrolled individuals, the mean BMI was 22.5 ± 3.3 kg/m^2^ and 31 (22.7%) had depression symptoms, while 14 (10.4%) had anxiety symptoms. According to the definition, 42 participants were categorized into the frailty group (30.7%) and 95 into the no-frailty group (69.3%). The frailty group showed worse performance on the MMSE (26.7 vs 28.0, *p* < 0.05) and MoCA (25.2 vs 26.1, *p* < 0.05) scores than the no-frailty group. The two groups also presented significant differences in age, education, comorbid conditions > 2, polypharmacy and depression. No significant differences were found between the two groups with respect to sex, BMI and anxiety symptoms (Table 1).

**Table 1:** Characteristics of 137 Participants Aged≥60 Years and Stratified by MBI Status and Frailty Status

Variable	Full Sample (*n* = 137)	MBI Status			Frailty Status		
		MBI–(*n* = 112)	MBI+ (*n* = 25)	*P*-value	No-frailty (*n* = 95)	Frailty (*n* = 42)	*P*-value
Age, mean (SD)	69.6 (7.6)	69.0 (7.5)	72.2 (7.7)	.05	67.9(6.9)	73.2 (8.1)	<.001
Female	94 (68.6)	86(76.8)	18 (72.0)	.61	58 (61.1)	36 (85.7)	.07
Education				.34			.01
Primary or lower	21 (15.3)	15 (13.4)	6 (24.0)		10 (10.5)	11 (26.2)	
Completed high school	75 (54.7)	64 (57.1)	11 (44.0)		50 (52.6)	25 (59.5)	
At least some college	41 (29.9)	33 (29.5)	8 (32.0)		35 (36.8)	6 (14.3)	
Comorbid conditions > 2	43 (31.4)	37 (33.0)	13 (52.0)	.08	27 (28.4)	23 (54.8)	.01
Polypharmacy	27 (19.7)	22 (19.6)	5 (20.0)	.97	9 (9.5)	18 (42.9)	<.001
BMI, mean (SD)	22.5 (3.3)	22.5 (3.2)	22.7 (3.9)	.75	22.8 (2.8)	21.9 (4.1)	.14
Depression (PHQ-9 ≥ 10)	31 (22.7)	26 (23.2)	5 (20.0)	.73	16 (16.8)	15(35.7)	.02
Anxiety (GAD-7 ≥ 10)	14 (10.2)	12 (10.7)	2 (8.0)	.69	8 (8.4)	6 (14.3)	.30
MMSE, mean (SD)	27.6 (2.4)	27.8 (2.3)	26.8 (2.9)	.049	28.0 (1.8)	26.7 (3.4)	.01
MoCA, mean (SD)	25.8 (2.5)	26.1 (2.2)	24.7 (3.2)	.009	26.1 (2.2)	25.2 (2.9)	.04

Notes: *SD* Standard deviation, *MBI* Mild behavioral impairment, *MBI-C* Mild Behavioral Impairment Checklist, *BMI* Body mass index; Results presented as n (%) unless otherwise noted. Chi-square tests were used for categorical variables, whereas t-tests were used for continuous variables

A total of 25 (18.2%) participants were MBI+, and 112 (81.8%) were MBI-. Regarding group composition, the mean age of MBI+ participants (72.2 ± 7.7) was higher than that of MBI– participants (69.5 ± 7.0) (*p* < 0.05). The MBI+ individuals had significantly poorer cognition, with lower MMSE (26.8 vs 27.8, *p* < 0.05) and MoCA (24.7 vs 26.1, *p* < 0.05) scores than the MBI- individuals. No significant differences were found between the MBI+ individuals and MBI- individuals in terms of sex, education level, BMI, comorbid conditions > 2, polypharmacy, or depression and anxiety symptoms (*p* > 0.05) (Table 1).

### Frailty and mild behavioral impairment

MBI status was significantly different between participants with and without frailty (*p* = 0.038). The MBI-C composite score was associated with frailty (*p* = 0.001). Of the five MBI domains, participants with decreased motivation, affective dysregulation and social inappropriateness MBI domains were more likely to have frailty. Neither impulse dyscontrol nor psychosis differed between frailty groups, although impulse dyscontrol neared statistical significance (*p* = 0.059) were found in our study (Table 2).

**Table 2:** Frailty and Cognitive and Behavioural Characteristics

	Frailty (*n* = 42)	No-frailty (*n* = 95)	χ2/F value	*ρ* value
MBI, n (%)	12 (28.6)	13 (13.7)	χ2 = 4.3	.038
MBI score, mean (SD)	7.3 (5.2)	4.7 (3.6)	F = 5.8	.001
Decreased motivation	2.2 (2.2)	1.3 (1.2)	F = 13.3	.005
Affective dysregulation	1.8 (1.4)	1.3 (1.1)	F = .6	.028
Impulse dyscontrol	2.3 (2.0)	1.6 (1.9)	F = 1.3	.059
Social inappropriateness	0.7 (1.0)	0.4 (.7)	F = 12.5	.041
Psychosis	0.3 (.7)	0.2 (.5)	F = 5.0	.246

*MBI* Mild behavioral impairment, *MBI-C* Mild Behavioral Impairment Checklist

Multivariable logistic regression analysis indicated that MBI+ status was significantly associated with higher risk of having frailty, with an OR of 3.09 (95% CI = 1.29–9.41; *p* = 0.047) (Table 3). We also evaluated the associations between frailty status and global cognition, depression, education and age; we found that age and depression were significantly related to a higher risk of having frailty (*p* < 0.05), but the association with education, MMSE and MoCA score was not significant (*p* > 0.05) (Table 3).

**Table 3:** Multivariable logistic regression analysis for the association between frailty status and objective cognition with mild behavioral impairment

	Frailty Status				
	β	Sr	Wal χ2	***ρ*** value	Odds ratio (95% CI)
Age	−.09	.04	5.28	.022	.91 (.84–.99)
Education	−.69	1.10	.40	.529	.50 (.06–4.3)
Depression	1.62	.51	10.27	.001	5.04 (1.88–13.58)
MoCA	−.13	.15	.73	.392	.88 (.66–1.18)
MMSE	.18	.16	1.21	.272	1.19 (.87–1.64)
MBI	1.13	.57	3.95	.047	3.09 (1.29–9.41)

*Abbreviations*: *CI* Confidence intervals

## Discussion

To our knowledge, this is the first cross-sectional study to evaluate the relationships between frailty, MBI, and cognition. First, we determined that frailty is common in this population, with a prevalence of 30.7%. Second, MBI was also fairly common, with a prevalence of 18.2%. Third, greater burden of frailty was associated with poorer cognition, measured using the MMSE (*p* = .01) and MoCA (*p* = .04). Fourth, compared to those without MBI, MBI+ status was associated with poorer cognition measured using the MMSE (*p* = .049) and MoCA (*p* = .01). Fifth, MBI+ status predicted higher levels of frailty (OR = 3.09; 95% CI = 1.29–9.41), and this signal was driven by the MBI domains of decreased motivation, affective/emotional dysregulation, and social inappropriateness (*p* < 0.05). These results suggest that in non-demented older adults, frailty and MBI are both common and associated with small but significant impairment in global cognition.

The prevalence of frailty was 30.7% in our study, which was relatively high compared with previous estimates, which ranged from 11% up to 26% in community samples [63–65]. This difference may be attributed to our study design and to the fact that participants came from primary care clinics. Frailty may increase the risk of future cognitive decline, and that cognitive impairment may increase the risk of frailty, suggesting that cognition and frailty may interact in the cycle of age-related decline [66, 67]. Our results indicated that frailty was associated with age-related cognitive decline, describing an at-risk group for the preclinical phase of neurocognitive disorders, consistent with previous studies [11–16]. In their seminal study, Solfrizzi and colleagues reported that frail older adults had a higher prevalence of cognitive impairment than those without frailty (77% vs. 54%) [68]. Furthermore, components of frailty appeared to be related to pathological findings of AD and vascular dementia, supporting the idea of a possible common biological pathway between frailty and cognitive disorders [69]. A previous study found that there was an increase in neurons with cellular senescence and aging of microglia, and therefore, increases in apoptosis, aggregation of protein, and mitochondrial dysfunction, with increased reactive oxygen species, oxidative damage to proteins and lipids, and accumulation of DNA damage [69]. Accordingly, increasing frailty may be an indicator of future cognitive impairment.

The prevalence of MBI (18.2%) in our participants was higher than that reported by Creese [22] in the PROTECT study, in which 10% of community-dwelling older adults aged 50 or over (*n* = 9931) reported MBI, as captured by the MBI-C. In a clinical sample of Spanish primary care patients from which the current cut-points were derived, the prevalence of MBI was 5.8% in older adults with subjective complaints [39] and 14.2% in MCI [40]. These estimates collectively, determined using the MBI-C, are considerably lower than previous prevalence estimated generated using the Neuropsychiatric Inventory [70] which ranged from 28 to 51% in a community population [71, 72], and 49–85% in a cognitive neurology clinic population [71, 73]. These differences may be due to the diagnostic frame of reference of 1 month of symptoms captured by the Neuropsychiatric Inventory, whereas the MBI-C involves a more rigorous standard of six-month symptom duration and explicit later-life onset of symptoms, in accordance with the MBI criteria. The lower MBI frequency generated using the MBI-C reflects increased diagnostic specificity for MBI, eliminating the inclusion of transient and reactive states, by excluding false positive symptoms.

Neuropsychiatric symptoms are associated with an increased risk of cognitive deficits across the lifespan, and MBI is associated with poorer cognition cross-sectionally [74], as well as longitudinally in comparison to those without MBI [23, 24]. In agreement with this previous evidence, we also found subtle but significant differences in global cognition reflected by lower scores on both the MMSE and MoCA in patients with MBI. Indeed, the MBI-C might have significantly higher discriminatory power than the MMSE when seeking to detect older adults with subtle cognitive decline [42]. Considering that MBI reflects the neurobehavioral axis of pre-dementia at-risk states and is a complement to the neurocognitive risk axis represented by MCI [31], this complementary approach may increase the yield when using both cognitive and behavioral approaches to screen for early-stage neurocognitive disorders.

In this study, we found that MBI was associated with higher levels of frailty, even after adjustment for potential confounders, and that this signal was driven by the MBI domains of decreased motivation, affective/emotional dysregulation and social inappropriateness. Our findings extend the literature by describing different patterns of association of MBI and its components with frailty, a pattern not previously established. Prior studies exploring the link between frailty and cognition have focused on individual functional abilities and assessed only global cognitive ability or limited cognitive domains [14, 75]. The mechanisms for the association are not clear, but possibly involve abnormalities in biological processes related to aging [76]. A growing body of epidemiological evidence indicates that the mechanisms involved in the onset of frailty are also those that promote neurodegeneration, including chronic inflammation [66] and oxidative stress [77]. Other clinical polypharmacy and multimorbidity can increase the risk of both frailty and dementia [78, 79].

MBI may serve as a proxy marker for frailty, or potentially a risk factor of frailty. Thus, MBI assessment may provide an approach to identify frailty early or to determine the risk of frailty in advance of completing a clinical assessment. This approach identifies potentially novel opportunities to prevent or delay frailty, age-related cognitive decline and other associated adverse health outcomes. The ease of administration of the MBI-C, which has been validated for telephone and online administration with high sensitivity and specificity [38, 39, 74], positions it as a simple and cost-effective tool to be administered remotely or at scale for detecting those at clinical risk, in order to flag them for further assessment and work up.

The limitations of our study include the participant population and the sample size. Lower prevalence of MBI and frailty among participants in communities rather than clinical, hospital, or institutional settings are to be expected, and it is unclear if these results can be generalized. We had a limited sample size in this study, and replication with a larger sample is required. Hence, the clinical utility of the cognitive frailty construct cannot be unequivocally supported by this study, but it should be further investigated in future studies independently undertaken by other investigators in older populations. The frailty instrument may also present another limitation. Due to the constraints related to time, resources, and space, we chose Fried phenotype, combining five physical and physiological burden items, determined simply and quickly. Additional studies with other multi-dimensional and more elaborate objective assessments, representing as many domains as possible, are needed in order to validate these findings.

## Conclusion

In conclusion, our findings provide further evidence that MBI and frailty are common among non-demented older adults, with both reflecting subtle but significant deficits in global cognition. MBI, especially in the domains of decreased motivation, affective dysregulation and social inappropriateness, is significantly associated with an increased risk of frailty in those with at most mild cognitive deficits. The MBI-C used in clinical practice could represent a simple and beneficial instrument for the detection of risk prior to the onset of frailty. Overall, these findings emphasize the importance of assessing physical as well as cognitive and behavioral function in older adults to identify risk. The inclusion of these measures in the assessment of frailty can improve the predictive validity of the phenotype regarding adverse health outcomes, and capture an at-risk group for early intervention.

## Data Availability

The datasets used and/or analyzed during the current study are available from the corresponding author on reasonable request.

## Abbreviations

MBI: Mild behavioral impairment
NPS: Neuropsychiatric symptoms
MCI: Mild cognitive impairment
AD: Alzheimer’s disease
NC: Normal cognition
MBI-C: Mild Behavioral Impairment Checklist
FP: The Fried phenotype
MoCA-BC: Chinese versions of the Montreal Cognitive Assessment
MMSE: Mini-mental State Examination
BMI: Body mass index
ANOVA: Analysis of variance
ORs: Odds ratios
CIs: Confidence intervals
